# A Comparison of Nifedipine Versus a Combination of Nifedipine and Sildenafil Citrate in the Management of Preterm Labour

**DOI:** 10.7759/cureus.42422

**Published:** 2023-07-25

**Authors:** Amber Hassan, Humaira Waseem, Nashwa AlDardeir, Hisham Nasief, Khalid Khadawardi, Ahmed B Alwazzan, Haneen Alothmani, Ziyad Hammad

**Affiliations:** 1 European School of Molecular Medicine, University of Milan, Milan, ITA; 2 Translational Neuroscience Lab, CEINGE-Biotecnologie Avanzate, Naples, ITA; 3 Research, Fatima Jinnah Medical University, Lahore, PAK; 4 Obstetrics and Gynecology, King Abdulaziz University Hospital, Jeddah, SAU; 5 Obstetrics and Gynecology, Umm Alquraa University, Makkah, SAU; 6 Oncology, King Abdulaziz University Hospital, Jeddah, SAU

**Keywords:** spontaneous preterm delivery, qtc prolongation, gestational age, preterm birth prevention, premature preterm labour rupture of membranes(pprom), s: preterm labour

## Abstract

Background

Preterm delivery is a significant contributor to neonatal and infant morbidity and mortality. Preventive methods are preferable to treatment protocols for reducing perinatal mortality and morbidity. The calcium channel blocker nifedipine has the potential to be employed as a tocolytic, whereas the phosphodiesterase inhibitor sildenafil citrate promotes smooth muscle relaxation.

Objective

This study aims to examine the tocolytic effect of nifedipine in combination with sildenafil citrate in managing preterm labour (PTL).

Methods

After approval from the ethical board, 160 patients fulfilling the selection criteria were enrolled in the study from the outpatient and emergency department of obstetrics and gynaecology, University of Lahore, Pakistan. After taking written informed consent, their demographic profile, i.e., name, age, gestational age at presentation, parity, and expected date of delivery was noted. Patients were randomly assigned in a 1:1 ratio to two study groups (Group A: sildenafil citrate + nifedipine) and (Group B: nifedipine) using a computer-generated random number table to obtain a trial sequence. In group A, each patient was given nifedipine 20 mg orally, followed by 10 mg orally every eight hours for 48 hours and vaginal administration of sildenafil citrate, 25 mg at eight-hour intervals, for 48 hours. In group B, females were given nifedipine 20 mg orally, followed by 10 mg orally every 8 hours for 48 hours. They were kept admitted for 72 hours. SPSS Statistics version 21.0 (IBM Corp. Released 2012. IBM SPSS Statistics for Windows, Version 21.0. Armonk, NY: IBM Corp.) was used to enter and analyse the collected data. Mean and standard deviation was calculated for quantitative variables like age, gestational age at presentation, gestational age at delivery, and BMI. Frequency and percentage were calculated for parity and preterm delivery.

Results

The study involved 160 patients, with the average age in Group A being 29.60±4.9 years and in Group B being 30.96±4.98 years. In terms of gestational age at delivery, Group A had an average of 34.16±1.7 weeks, while Group B had an average of 33.5±1.8 weeks (p-value<0.05). Preterm delivery was observed in 68.5% of Group A and 41.3% of Group B, with a significant p-value of 0.001. The study also discovered that the duration of prolonged pregnancy was significantly higher in Group A compared to Group B, with averages of 14.96±10.37 days and 10.24±8.97 days, respectively (p-value=0.002).

Conclusion

The results of this study suggest that the combination of sildenafil citrate and nifedipine may offer a promising new approach to improving pregnancy outcomes in cases of PTL. In the present study, sildenafil citrate plus nifedipine showed a significant effect in the management of PTL and prolongation in mean gestational age at delivery.

## Introduction

Preterm labour (PTL) is regular palpable uterine contractions resulting in cervical dilatation and effacement before the completion of 37 weeks of pregnancy [1]. During PTL, the purpose of tocolytic is to postpone delivery to get time for administration of corticosteroids in addition to magnesium sulfate (24-34 weeks) to reduce the incidence of respiratory problems and to arrange patient transfer to tertiary care with a good neonatal intensive care unit (NICU) [2].

Preventing premature births continues to be one of the most difficult issues for obstetricians around the world, primarily to prevent complications from neonatal prematurity that cause short- and long-term morbidities. Prematurity prevention will also lower the incidence of neonatal mortality [3]. Treatment with calcium channel blockers such as nifedipine inhibits uterine contraction in PTL at a higher rate compared with placebo by reducing calcium influx into the cells. Nifedipine is the first-line drug for tocolytic according to the Royal College of Obstetricians and Gynaecologists guidelines [4].

Oxytocin receptor antagonists can be given in case of nifedipine contraindication. The combination of tocolytic drugs (nifedipine with other tocolytic agents) resulted in a significantly greater inhibitory effect on contractility than each drug alone [5,6]. In separate studies, sildenafil was demonstrated to relax isolated pregnant human myometrium. The first of these two studies discovered that sildenafil caused dose-dependent relaxation of the myometrium that was not reversed by the guanylyl cyclase inhibitor methylene blue or the nonspecific potassium channel blocker tetraethyl ammonium (TEA) at 5 and 10 mM concentrations. At a dosage of 20 mM, TEA reversed the relaxant action of sildenafil. These data revealed to the authors that potassium channels are likely to mediate sildenafil's relaxing impact on the myometrium [7,8].

Sildenafil is a smooth muscle relaxant that inhibits phosphodiesterase-5, the enzyme that catalyses the metabolism of the second messenger cyclic GMP. The primary cGMP receptor is protein kinase G, which promotes relaxation by acting on Ca2+-activated K+ channels (BKCa) [9-11]. Increased cGMP levels promote muscle relaxation by reducing intracellular calcium levels. El-Aziz et al. found sildenafil and nifedipine combination superior to nifedipine in PTL and are associated with a reduction in NICU admission, improved birth weight, and fewer neonatal complications [12]. Another study found sildenafil citrate and nifedipine combination a better option for tocolytic therapy of threatened PTL [13].

The rationale of this study is the comparison of the tocolytic effects of nifedipine alone and in combination with sildenafil citrate in the management of PTL in our setting. In addition, not much work has been done in this regard and few studies found regarding such trials. This will help us to improve our practice in future for better management of PTL.

## Materials and methods

Data collection procedure

After approval from the ethical review committee of the University of Lahore, Pakistan, the 160 females with singleton gestation presenting at gestational age 30 weeks to 34 weeks with PTL and cervical dilatation of ≤ 3 cm patients were enrolled in the study from the outpatient and emergency department of obstetrics and gynaecology, the University of Lahore Teaching Hospital, Lahore, Pakistan. Non-probability consecutive sampling was used to enroll the patients, and they were randomly allocated to both groups using the balloting method.

Demographic profile and group assignment

After taking written informed consent, their demographic profile, i.e., name, age, gestational age at presentation, parity, and estimated date of delivery was noted. It was an open-label randomised controlled trial according to CONSORT guidelines. Enrollment and observation along with continuation of medication were done by residents of equal experience. Patients were randomly assigned in a 1:1 ratio to two study groups (Group A: sildenafil citrate + nifedipine) and (Group B: nifedipine) using a computer-generated random number table to obtain a trial sequence which was hidden in a sealed number opaque envelope. Each envelope contains an assignment for a single patient. The biostatistician generated the random allocation sequence, and the investigator enrolled the participants.

At the time of admission, high vaginal swabs and midstream urine were preferred for cultural sensitivity. For conservative management of preterm prelabour rupture of membranes, adjunctive antibiotic treatment is recommended. Ascending infections can be treated with these antibiotics. The use of erythromycin, either with or without ampicillin/amoxicillin, has been linked to several benefits for maternal and neonatal.

In Group A, patients were given nifedipine 20 mg orally, followed by 10 mg orally every eight hours for 48 hours and vaginal administration of sildenafil citrate (25 mg at eight-hour intervals) for 48 hours. In Group B, patients were given nifedipine 20 mg orally, followed by 10 mg orally every eight hours for 48 hours. They were kept admitted for 72 hours.

Examination and monitoring

Maternal (pulse rate, blood pressure, temperature, uterine contractions) and fetal monitoring (fetal heart rate) were performed during a hospital stay. Uterine contractions were observed clinically for one hour in terms of frequency (number of palpable contractions in 10 minutes), duration in seconds, and strength (mild, moderate, strong). A pelvic examination was done after four hours to assess cervical dilatation if palpable uterine contractions persist.

Drug administration and patient response

All patients received two doses of Injection dexamethasone (for fetal lung maturity), 12 mg intramuscular in the gluteal region, with intervals of 12 hours, unless given previously. Patients were kept in the first-stage labour ward. During 72 hours of hospital stay, all the patients were observed for side effects of both drugs and if any one of them develops an allergic reaction, hypotension (systolic blood pressure less than 90 mmHg), flushing, headache, tachycardia, (nifedipine) allergic reaction, gastritis, headache, dizziness, (sildenafil citrate). If cervical dilatation was more than 3 cm, treatment failure was labelled, and patients were given treatment accordingly. If the patient responds to treatment and uterine contractions are suppressed during 48 hours, the primary outcome was achieved. Patients were observed for the next 24 hours and were discharged from there. Their contact number and residential address were noted. Patients who did not deliver in our setting hospital were contacted telephonically. None of these medicines was prescribed on discharge, and patients were advised to continue their routine medications. Patients were followed every one week till delivery. Patients were educated about the sign and symptoms of labour (painful uterine contractions, rupture of membranes, per vaginal bleeding), and they were advised to report to the labour room in case of labour pains, and the date and time of delivery were noted. The secondary outcome was the prolonged duration of pregnancy. All this information was recorded on proforma (attached).

Statistical analysis

SPSS Statistics version 21.0 (IBM Corp. Released 2012. IBM SPSS Statistics for Windows, Version 21.0. Armonk, NY: IBM Corp.) was used to enter and analyse the collected data. Mean and standard deviation for quantitative variables like age, gestational age at presentation, gestational age at delivery, and BMI were calculated. Frequency and percentage were calculated for parity and preterm delivery. The comparison of proportions of PTL managed (yes/no) and preterm delivery (yes/no) was done by the chi-square test. The mean duration of management of PTL (hours) and duration for which pregnancy is prolonged (days) were compared by independent sample t-test according to the normality of data. A p-value of ≤0.05 was taken as significant.

## Results

The study included a total of 160 patients, with a mean age in Group A of 29.60±4.9 years and in Group B of 30.96±4.98. At presentation, the patients had a mean gestational age of 32.03±1.32 weeks in Group A and 32.04+1,39 in Group B. The mean BMI of the patients was 26.22±1.59 kg/m2 in Group A as compared with Group B at 26.27±1.73 kg/m2 as shown in Table 1. Out of the 160 patients, 15 (18.8%) were nulliparous in Group A and 18 (22.5%) in Group B. Both groups did not have any significant differences between them concerning age, gestational age at presentation, BMI, and parity (p-value of >005), as shown in Table 1.

**Table 1:** Baseline characteristics of the study sample Independent sample t-test/chi-square test, p-value of <0.05*, Group A: sildenafil citrate + nifedipine, Group B: nifedipine

	Study Groups	Mean	Std. Deviation	P-value
Age	Group A	29.60	4.9	0.084
	Group B	30.96	4.98	
Gestational Age at Presentation	Group A	32.03	1.32	0.954
	Group B	32.04	1.39	
BMI	Group A	26.22	1.59	0.846
	Group B	26.27	1.73	
Gestational Age at Delivery	Group A	34.16	1.76	0.020*
	Group B	33.50	1.80	
Parity Nulliparous f(%)	Group A	15	18.8%	
	Group B	18	22.5%	0.558
Multiparus f(%)	Group A	65	81.3%	
	Group B	62	77.5%	

**Figure 1:**
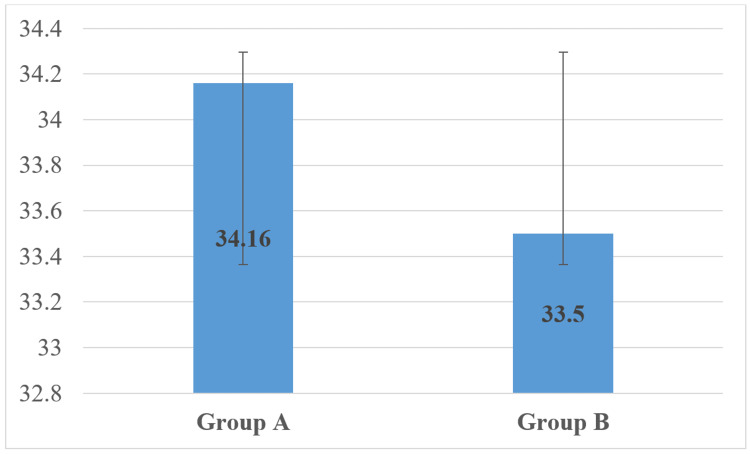
Comparison of gestational age at delivery Independent sample t-test p-value of <0.05*

The mean gestational age at delivery in Group A was 34.16±1.7, while in Group B, it was 33.5±1.8 weeks. The difference was statistically significant, with a p-value of 0.020 (Figure 1).

The study found that postterm pregnancy was significantly higher in Group A than in Group B (14.96±10.37 vs. 10.24±8.97; p-value=0.002), as shown in Table 2.

**Table 2:** Comparison of postterm pregnancy

Comparison of Days Delivery Prolonged	Study Groups	Mean	Std. Deviation	P-value
	Group A	14.96	10.37	0.002
	Group B	10.24	8.97	

**Figure 2:**
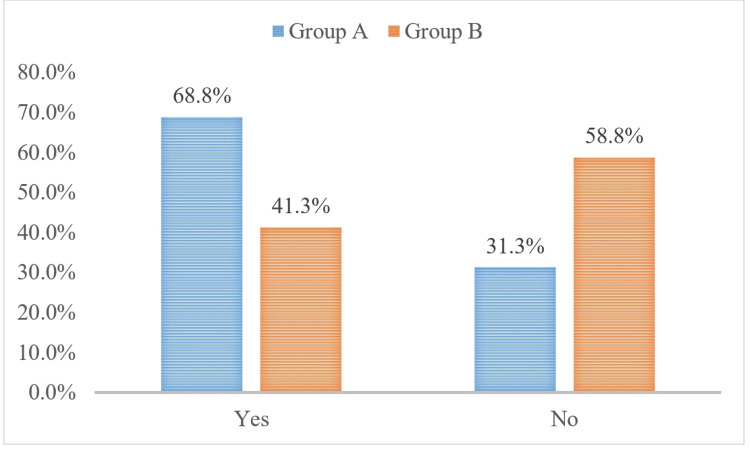
Preterm delivery within 48 hours chi-square test, p-value of =0.001

Preterm delivery, where it was prolonged beyond 48 hours, was 68.5% vs. 41.3% in Group A and B, respectively, with a p-value of 0.001, as shown in Figure 2.

The study also compared the neonatal outcomes between the two groups. The incidence of NICU admission was 3.8% vs. 8.8% in Group A and B, respectively, while mortality was 2.5% vs. 6.7% in Group A and B, and RDS was reported as 13.4% vs. 9.4% in Group A and B, respectively, with a p-value of 0.324. The incidence of flushing was 3.8% vs. 12.5% in Group A and B, respectively, with insignificant association (Table 3).

**Table 3:** Fetal outcomes among groups NICU: neonatal intensive care unit Chi-square test, P-value of <0.05*

Variables		Group A	Group B	P-value
Admitted to NICU	Yes	3(3.8%)	7(8.8%)	0.193
	No	77(96.3%)	73(91.3%)	
Neonatal Mortality	Yes	2(2.5%)	6(7.5%)	0.147
	No	78(97.5%)	74(92.5%)	
Respiratory Distress Syndrome	Yes	3(3.8%)	10(12.5%)	0.43
	No	77(96.3%)	70(87.5%)	

## Discussion

PTL is a highly important subject in obstetrics and gynaecology due to the potential long-term psychological impact on families associated with caring for premature infants [14]. The primary objective of pregnancy is the delivery of a healthy baby at term, and although PTL cannot be stopped, it can be postponed for a few days. The outcomes of preterm delivery, such as infant death and impairment, may be significantly impacted by this delay. The calcium channel blocker nifedipine, which has a quick onset and brief duration of action, is a prototype for dihydropyridine. It works by blocking the voltage-dependent L-type calcium channel, which reduces the amount of calcium that enters cells, relaxes smooth muscles, and has detrimental inotropic and chronotropic effects on the heart. Indirect cardio-stimulation is caused by vasodilation, which is followed by a sympathetic tone rise mediated by baroreceptors. As an anti-hypertensive drug, it is marketed. The oral administration route, the availability of instant and slow-release preparations, the low incidence of side effects (mild), and its reasonable cost all contribute to the rapid and extensive diffusion of this medication in the obstetric sector [15]. For many years, men with erectile dysfunction have utilised sildenafil. By inhibiting phosphodiesterase and acting on nitric oxide, this medication exhibits potent vasodilator effects. This medicine has been studied for the treatment of uterine umbilical artery problems and uterine muscle contractions, and it has enhanced vascular exchange on the one hand and modulated uterine contractions on the other [16]. It has now been brought up that sildenafil is increasingly being used to treat vascular or contractile issues in pregnant women. The effectiveness of the combined protocol of nifedipine and sildenafil in treating PTL and delaying delivery was assessed in the current trial in comparison to nifedipine alone.

The mean age of the patients in this study was 30.06±4.939 years, and a similar mean age of such patients was previously reported by Qurat-Ul-Ain et al. as 30.09±4.11 years [9]. These differences in gestational age may be attributed to variations in study populations, sample sizes, and study designs. The mean BMI of the patients in this study was 26.28±1.601 kg/m2, which is slightly lower than the mean BMI of 30.09±3.05 kg/m2 reported by Nasrolahei et al. [17]. This difference may be due to variations in study populations and geographic locations. The study sample included 54 (21.3%) nulliparous participants and 200 (78.7%) multiparous participants. This finding is consistent with other studies, which reported nulliparous rates of 10.8% vs. 89.2% and 11.4% vs. 88.6%, respectively. The higher rate of multiparous participants may be attributed to the fact that this group has a lower risk of PTL compared to nulliparous women. Overall, these findings provide important demographic information about the study population and offer a basis for comparison with other studies. Understanding the characteristics of the study population is critical for interpreting the results and generalising the findings to other populations. This difference may be associated with the sample section criteria.

The study found a significant difference in mean gestational age at delivery between Group A and Group B. The mean gestational age at delivery was higher in Group A (34.51±1.830 weeks) compared to Group B (33.60±1.774 weeks), with a p-value of 0.020 These findings suggest that the addition of sildenafil citrate to nifedipine may be effective in prolonging gestational age in women experiencing PTL. This finding is consistent with previous studies that have also reported a significant difference in gestational age at delivery between groups. The increase in gestational age at delivery is important as it can lead to improved outcomes for both the mother and the baby. For instance, it can reduce the risk of neonatal morbidity and mortality, and increase the chances of a successful delivery. Therefore, the findings of this study provide valuable insights into the management of PTL and may inform clinical practice guidelines in the future.

The study found a significant difference in the number of days that pregnancy was prolonged between Group A and Group B. Group A had a mean prolongation of 17.58±10.65 days, while Group B had a mean prolongation of 10.58±8.99 days, with a p-value of 0.001. These findings suggest that the addition of sildenafil citrate to nifedipine may significantly prolong pregnancy in women experiencing PTL. Similar results were reported, indicating a mean prolongation of 16.17±5.14 days in the group receiving sildenafil citrate and nifedipine compared to 9.98±3.58 days in the group receiving nifedipine alone, with a p-value of 0.001. However, other studies have reported even higher mean prolongations in pregnancy. Overall, the findings of this study suggest that the addition of sildenafil citrate to routine hydration therapy may be an effective strategy for prolonging pregnancy in women experiencing PTL, which may lead to improved maternal and fetal outcomes. These findings have important implications for clinical practice and may inform future guidelines for the management of PTL.

Delivery within 48 hours of admission was 20.5% vs. 32.3% (p-value of =0.033), and the same factor constituted efficacy of the study where efficacy between the groups in preventing delivery for 48 hours was 79.5% vs. 67.7% (a p-value of =0.033). Previous studies also support findings in this study where this relation was reported as 82.9% vs. 70.5% (p-value of 0.005), 92.5% vs. 82.5% (p-value of =0.005), and 81.8% vs. 86.6% (p-value of =0.018), as reported by various studies. The study compared the incidence of complications and adverse reactions between two groups, Group A and Group B. Group A had a higher incidence of allergic reactions compared to Group B (5.5% vs. 4.7%), but the difference was not statistically significant (p-value of =0.355). Similarly, the incidence of headaches was higher in Group A compared to Group B (19.7% vs. 11.8%), but the difference was not statistically significant (p-value of =0.085). The study compared the incidence of flushing between two groups, Group A and Group B. The results showed that 11.8% of participants in Group A reported flushing, while only 7.1% of participants in Group B reported the same. However, the difference between the two groups was not statistically significant, with a p-value of 0.198.

The term "hypotension" refers to abnormally low blood pressure, which can lead to symptoms such as dizziness, lightheadedness, and fainting. The study mentioned compares the incidence of hypotension in two groups of participants, Group A and Group B, who likely received different treatments or interventions. The study found that the percentage of participants who experienced hypotension was higher in Group A (2.4%) than in Group B (1.6%), but the p-value associated with this difference was 0.355, indicating that it was not statistically significant. A p-value is a measure of the strength of evidence against the null hypothesis, which states that there is no difference between the two groups being compared. In general, a p-value less than 0.05 is considered statistically significant, meaning that the observed difference between the groups is unlikely to be due to chance alone. Another study conducted by El-Aziz et al. also investigated the incidence of hypotension in two groups of participants: one receiving sildenafil citrate and nifedipine and the other receiving nifedipine alone. The study found that a higher percentage of participants in the sildenafil citrate and nifedipine group (5.0%) experienced hypotension compared to the nifedipine alone group (2.5%), but the p-value associated with this difference was 0.556, indicating that it was not statistically significant. Overall, while both studies found differences in the incidence of hypotension between groups, the p-values indicate that these differences were not statistically significant. It is important to consider other factors that may have influenced the results, such as the sample size, the specific interventions used, and the characteristics of the participants. These findings suggest that the addition of sildenafil citrate to nifedipine does not significantly increase the risk of adverse effects such as flushing or hypotension. However, clinicians need to monitor patients closely for any potential adverse effects and adjust treatment accordingly. Overall, the addition of sildenafil citrate to routine hydration therapy appears to be a safe and well-tolerated strategy for managing PTL.

One of the biggest strengths of the study was the fairly good sample size, which indicates that a sufficient number of participants were included in the study. Additionally, the study used strict inclusion and exclusion criteria, which can help to ensure that the sample is as representative and unbiased as possible. The strength of the study was that all of the patients were managed by the same staff, which could help to reduce bias and ensure consistency in the treatment and data collection processes. However, the study also had some limitations. For example, it was a single-centre study, meaning that all of the participants were recruited from one location. This can limit the generalizability of the findings to other populations or settings. Additionally, the study had time constraints, which could have limited the amount of data that could be collected or analysed. To address these limitations and to gain a more comprehensive understanding of the topic being studied, the authors suggest that future research should involve larger, multicentre trials. These types of studies can help to increase the sample size and diversity of the participants and can also help to provide more detailed information about potential complications or other outcomes of interest.

## Conclusions

The present study investigated the efficacy of combining sildenafil citrate and nifedipine in the management of PTL. The results of the study demonstrated that combination therapy was significantly more effective in prolonging the duration of pregnancy beyond 48 hours compared to treatment with nifedipine alone. Moreover, the mean gestational age at delivery was also significantly higher in the group treated with sildenafil citrate and nifedipine, indicating a more successful pregnancy outcome. The results of this study are promising, as they suggest that combining sildenafil citrate with nifedipine may be an effective treatment option for PTL. By prolonging the duration of pregnancy beyond 48 hours, this therapy may allow for the administration of corticosteroids to improve fetal lung maturity and magnesium sulfate for fetal neuroprotection may improve neonatal outcomes and reduce the risk of respiratory distress syndrome. Overall, the findings of this study are significant as they provide evidence for a treatment option. Further research is needed to confirm these findings and establish the safety and efficacy of sildenafil citrate in the management of PTL.
